# Event-related potentials to changes in facial expression in two-phase transitions

**DOI:** 10.1371/journal.pone.0175631

**Published:** 2017-04-13

**Authors:** Michael J. Wright, Lisa K. Kuhn

**Affiliations:** 1 Centre for Cognitive Neuroscience, Department of Life Sciences, College of Health and Life Sciences, Brunel University London, Uxbridge, United Kingdom; 2 Experimental Neuropsychology Unit, Department of Psychology, Saarland University, Saarbrücken, Germany; Leiden University, NETHERLANDS

## Abstract

The purpose of the study was to compare event-related potentials (ERPs) to different transitions between emotional and neutral facial expressions. The stimuli contained a single transition between two different images of the same face, giving a strong impression of changing expression though apparent motion whilst eliminating change in irrelevant stimulus variables such as image contrast or identity. Stimuli were calibrated for intensity, valence and perceived emotion category and only trials where the target emotion was correctly identified were included. In the first experiment, a magnification change (zoom) was a control condition. Transitions from neutral to angry expressions produced a more negative N1 with longer peak latency, and more positive P2 than did an increase in magnification. Critically, response to neutral following angry, relative to neutral following magnified, showed a generally more negative ERP with a delayed N1 peak and reduced P2 amplitude. In the second experiment, comparison of neutral-happy and neutral-frightened transitions showed significantly different ERPs to emotional expression change. Responses to the reversed direction of a transition (happy-neutral and frightened-neutral) were much reduced. Unlike the comparison of angry-neutral with magnified-neutral, there were minimal differences in the responses to neutral following happy and neutral following frightened. The results demonstrate in a young adult sample the directionality of responses to facial expression dynamics, and suggest a separation of neural mechanisms for detecting expression changes and magnification changes.

## Introduction

Facial expressions change: they are intrinsically dynamic. All facial expressions arise from preceding expressions, and an emotional expression will often arise from a neutral facial expression and at some point return to an emotionally neutral expression, so what is needed initially is an understanding of the transitions from neutral to emotional and from emotional to neutral. Several studies have shown that recognition of emotional expressions is enhanced in dynamic relative to static pictorial presentations, particularly for subtle or low intensity facial expressions [1–5].

Regarding the neural basis for processing dynamic facial expressions, Haxby, Hoffman and Gobbini [6] proposed that, after initial visual perception of faces in the inferior occipital gyri (occipital face area: OFA), the fusiform face area (FFA) mediates the perception of relatively unchanging aspects of faces such as identity and gender, whereas the superior temporal sulcus (STS) mediates the perception of changeable aspects such as facial expression and gaze. STS may also provide a secondary route to face recognition via dynamic facial signatures [7]. The amygdala has been identified as an additional neural element in a network for processing socially relevant actions [8] and further extensions and modifications to the Haxby, et al. model [6] have been proposed [9–11]. Moreover a recent critical review [12] confirmed the primary division of the face processing system between a form-sensitive ventral stream passing through FFA and a motion-sensitive dorsal stream passing through STS. If initial structural face encoding and later emotion encoding are embedded in anatomically segregated systems [6, 11], investigating changes to emotional expression should tap into the system that processes changeable aspects of faces in the STS as compared to the perception of early face signals in the OFA and stable face attributes in the FFA.

Electroencephalography (EEG) can contribute information about the temporal dynamics of the face processing network. Early event-related potential (ERP) studies of facial expression generally used pictorial stimuli appearing abruptly out of a blank background, that is, face onset rather than the onset of a facial expression. Such stimuli elicit a prominent negative potential, the N170, which is larger in amplitude for stimuli perceived as faces than for non-faces, over right lateral occipital-temporal locations, and is accompanied by a frontal-central positivity. Several studies have found differences in ERP waveforms [13] or peak latencies [14] for different categories of emotional facial expression. Other studies have found substantial differences between ERPs for emotional and neutral faces but similar responses to different emotion categories [15, 16]. A meta-analysis of 57 experiments [17] concluded that the greatest enhancement of N170 relative to neutral expressions was produced by anger, followed by fear and then happiness, but a minority of the studies reviewed found significant N170 differences in direct comparisons of ERPs to different emotions. These ERP studies to onset of stationary expressions are numerically dominant in the literature and ERP studies of the dynamics of facial expressions are relatively few.

Work by Recio and collaborators [5, 18] explored the neural basis of dynamic facial expressions using ERP, identifying enhanced and prolonged early posterior negativity (EPN) and late positive complex (LPC) in dynamic displays. The EPN was responsive to the intensity of non-emotional as well as emotional facial movements, and showed little difference in the response to different emotions; however the LPC was larger for negative than positive emotional expressions [18]. The dynamic stimuli used [18] progressed within 150 ms from onset of a neutral face to development of an emotional expression.

Miyoshi, Katayama and Morotomi [19] used two-phase stimuli comparing happy and neutral expressions, and demonstrated an enhanced N1 to the transition from neutral to happy, compared with a change in facial identity or to an expression plus identity change or to a change with inverted faces. Miki et al. [20] also utilised two-phase stimuli, and found differences between children and adults in a posterior temporal negative wave, with shorter peak amplitudes and latencies found in adults compared to children.

Using two-phase transitions allows to test whether the ERP is sensitive to the direction of change. The emotional meaning of such transitions is directionally sensitive: for example a change from a fearful to neutral has a different emotional meaning than a change from neutral to fearful. Indeed, Miki et al. [20] reported directional effects in children’s ERPs with larger peak amplitudes at temporal-posterior electrodes for changes from neutral to emotional as compared to changes from emotional to neutral. However, they reported only small directional differences in a young adult control group when compared to children. Also, Miki et al. [20] used a secondary task (to press a button when the eyes in the face were averted) to distract attention away from the facial emotion, whereas the present study emphasises the importance of correct identification of the target emotion to ensure depth and consistency of processing.

### Aims of the study

The first aim of the present experiments was to compare the ERP to different types of transitions in facial expressions occurring 500 ms after onset of the first expression, when the early ERPs to face onset have completed. Experiment 1 examined whether the response to a dynamic transition in emotional expression (from neutral to angry) differed from apparent motion (looming) of a neutral face. Importantly, it also measured whether the response to a neutral face differed according to whether the preceding facial expression was an angry or a magnified image of the same face. Experiment 2 tested whether the ERP transition from neutral to a happy expression differed from that from a neutral to frightened expression. It also tested for differences in the ERP to the reverse-order transition, comparing the response to neutral preceded by happy with neutral preceded by frightened expressions.

A second aim of the present study was to replicate the finding of directional emotion effects in ERPs of Miki et al. [20] in a larger young adult sample.

Four hypotheses were tested for each experiment: for experiment 1. H1.1: The ERP to neutral-angry transitions differs from the ERP to neutral-magnified transitions. H1.2: The response to angry-neutral transitions differs from the response to magnified-neutral transitions. H1.3: The ERP to neutral-angry transitions differs from the ERP to angry-neutral transitions; H1.4: The ERP to neutral-magnified transitions differs from the response to magnified-neutral transitions. A similar set of hypotheses was tested in experiment 2. H2.1: The ERP to neutral-frightened transitions differs from the ERP to neutral-happy transitions. H2.2: The ERP to frightened-neutral transitions differs from the ERP to happy-neutral transitions. H2.3: The ERP to neutral-frightened transitions differs from the ERP to frightened-neutral transitions. H2.4: The ERP to neutral-happy transitions differs from the ERP to happy-neutral transitions.

## Materials and methods

### Participants

Participants were university students with an age range from 18 to 21 and the sample included participants of European, Asian and African ethnicity. EEG data was analyzed from 19 out of 20 participants for Experiment 1 (*M* age 19.3, 13 female 7 male) and from 16 out of 21 participants for Experiment 2 (*M* age 19.5, 10 female 6 male). Six out of 41 EEG recordings were excluded across the two experiments, two due to movement artefacts and four due to incorrect filter settings during acquisition.

Separate groups of students took part in rating studies to calibrate the stimuli (see S1 File). All participants had normal visual acuity and wore their prescription glasses or contact lenses during the experiment if required.

### Ethical statement

The experimental protocol was approved by the Psychology Research Ethics Committee before initiating the study. It followed the University’s guidelines for research ethics and was in accordance with the Declaration of Helsinki. After a full briefing on the experimental procedures, participants gave their written informed consent, and were informed of their right to withdraw from the study at any time without adverse consequences, academic or otherwise.

### Stimuli: Validation studies

Face stimuli were pairs of greyscale images taken from the Japanese Female Facial Expressions (JAFFE) dataset [21]. Face stimuli were categorised and rated singly by a student sample separate from those in the main EEG study, and scores for JAFFE stimuli were compared with those for female faces in the validated Ekman and Friesen dataset [22]. Details of the calibration of source pictures are given in a supplement to this paper [S1 File]. We concluded that the JAFFE dataset is comparable with Ekman and Friesen’s but with lower intensities of facial expression.

Behavioural data gathered during EEG recording showed comparably high rates for identification of the target emotion with dynamic stimuli. For this reason, it was possible to ensure that ERP data analyses for JAFFE faces were restricted to trials on which the target emotions were correctly identified. However the question also arises as to whether emotions are differently perceived for static versus dynamic presentation of the stimuli [23]. In order to test this possibility with the two-phase dynamic stimuli used in the present study, a series of post-hoc behavioural experiments was carried out separate from the main study. These experiments are also described in the supplementary file [S1 File].

### Stimuli: Rationale and description

The simplest possible dynamic change is a transition between two states, and an analysis of responses to two-phase stimuli has provided some fundamental insights into perceptual processes [24]. Although it is less realistic than continuous change, an instantaneous change provides an unambiguous temporal reference for ERP averaging and thus for the analysis of latency as well as amplitude of ERP waveforms.

The ERP to a change from a neutral to an emotional expression is likely to show a better signal-noise ratio specific to the emotional expression than the ERP to the onset of an emotional face, because the preceding neutral expression serves as an inbuilt control for emotion-irrelevant stimulus variables such as image contrast and colour, general shape and type of object, gender and identity. Electrophysiological studies of gaze processing and other non-emotional facial movements have always taken a dynamic approach and have shown that facial movements such as changes in gaze direction elicit specific ERPs [25].

In the present ERP experiments, images of ten individuals were presented in pairs, each image of a pair representing a different expression of the same individual’s face. Each image was presented for 0.5 s and the second image followed the first without a gap. Co-registration of the images produced strong apparent motion of facial features such that each image pair gave an impression of an abruptly changing facial expression. Stimuli were presented on a Vision Master Pro CRT monitor driven at 100Hz frame rate by a VSG2/3 visual stimulus generator (Cambridge Research Systems). ImageJ [26] was used to resize, and to measure average intensities and pixel-wise image differences. There was a grey screen between trials, and presentation order was randomized. The mean luminance of the inter-stimulus screens and the picture surrounds was matched to that of the pictures in order to reduce irrelevant visual stimulation. Experiment 1 compared the response to face pairs containing an emotional expression change with control pairs that contained a magnification change (zoom). The magnification change was 10% in area, which approximated the mean proportion of pixels (averaged across the 10 stimuli in the set) whose intensity changed when moving from an angry to a neutral face (or vice versa). A magnification change was chosen as the control because many aspects of face perception are robust over changes in magnification [27, 28]. Experiment 2 compared the response to transitions between neutral and two different facial expressions, happy and frightened.

### Procedure and task

Participants signed a consent form and then filled in a short questionnaire on demographic details. Then the electrode cap was fitted and the electrodes filled with gel. Participants were then seated in front of the stimulus screen and given a hand-held button box. After impedance testing, a sample EEG was recorded, then the room lights were dimmed and the experiment began. A brief practice trial was shown in order to explain the general nature of the stimuli and the task, together with instructions to minimize movement and maintain concentration during the experimental blocks. The task in Experiment 1 was to indicate whether the trial indicated a change in expression or a change in magnification. The task in Experiment 2 was to report whether the sequence contained a happy or a frightened expression. The participants responded to face stimuli with a button press when prompted by an auditory cue presented 1.8 s, 2.0 s or 2.2 s (randomized) after the end of the second face image. The response was delayed in order to minimize overlap of stimulus- and response-related ERPs. At 2 s after the auditory cue the next face pair was presented whether or not a response had occurred. Generally five blocks of 100 trials were run, each lasting approximately 8.5 min, with rest intervals between them.

### EEG recording

Participants wore a 32 channel Quik-cap with sintered ceramic Ag/AgCl electrodes filled with Quik Gel (Compumedics Neuromedical Supplies). Electrodes were located at the following 10/20 positions: O1, Oz, O2, P7, P3, Pz, P4, P8, TP7, CP3, CPz, CP4, TP8, T7, C3, Cz, C4, T8, FT7, FC3, FCz, FC4, FT8, F7, F3, Fz, F4, F8, FP1, and FP2. An isolated ground was placed at AFz and an average mastoid reference was used. Vertical and horizontal electrooculogram (EOG) were also recorded. Impedances were < 10 KΩ. EEG was amplified at a gain of 1000 and bandpass 0.15–100 Hz with 50Hz notch and digitized at 1000 Hz using a Synamps amplifier and Scan 4.2 acquisition and analysis software (Compumedics Neuroscan).

### Data analysis

The offline EEG time series was low pass filtered at 30 Hz, 24dB/octave, with zero phase shift. Blink artifacts were removed from the EEG channels by a principal components method. The cleaned EEG time series was epoched from -1100 to 1000 ms such that 0 ms was the onset of the second stimulus image. Sweeps were baseline corrected, and those containing EEG amplitudes greater than ±75 μV were rejected. Average ERPs were obtained for all four conditions for each participant.

As there is incomplete knowledge concerning the waveforms and scalp distributions of dynamic facial expression ERPs, a mass univariate approach was adopted for data analysis [29]. The data range for the univariate analysis was 0–500 ms, across all electrodes, down-sampled using spline interpolation to a frequency above the Nyquist rate for the filtered EEG (15 ms/sample). ERP differences between experimental conditions were determined for each participant, and the paired-sample *t*-statistic for the group data was calculated for these differences. The significance level of the *t-*values was corrected for multiple comparisons (electrodes x time samples) using a false discovery rate (FDR) correction [30].

The main conclusions from the univariate analysis were confirmed and extended by ANOVA on ERP amplitudes and latencies in predefined regions of interest (ROIs). The two methods of statistical inference are complementary: the univariate approach requires no prior hypotheses about the timing and location of responses to the experimental conditions, and includes a correction for multiple comparisons. The ANOVA approach has greater sensitivity but achieves this at the cost of requiring predefinition of temporal and spatial regions of interest, so it could miss effects if these were not defined optimally. Convergence of results from the two approaches would increase confidence that findings are robust. The principal data analysis was performed on the response to the transition and for this purpose the average ERP epoch was cut to -100 ms to 500 ms. Supplementary data were analyzed for ERPs to the first frame, that is, to face onset. Prior to amplitude and latency measurements, the average ERPs to the transition were again baseline corrected to a peri-stimulus interval (-10 to +10 ms). Amplitudes and latencies were detected in the individual average ERP data for each experimental condition by a peak detection algorithm (Scan 4.3) operating across all electrodes within specified time windows and referenced to the relevant event (face transition to the second interval): 70–120 ms for P1, 140–200 ms for N1, 190–260 ms for P2, 250–350 ms for EPN [18] and 300–500 ms for LPC [18]. Spatial ROIs used were occipital-parietal: P7, O1, Oz, O2, P8, parietal: P7, P3, Pz, P4, P8, or midline: Oz, Pz, CPz, Cz, FCz, Fz. P1 is thought primarily to reflect visual processing in posterior cortex, so the occipital-parietal electrodes were selected. In accordance with the reported scalp distribution of N170, parietal electrodes P7, P3, Pz, P4 and P8 were chosen as the ROI for N1. P2 and LPC typically show widespread distributions with maxima in parietal, central or frontal regions therefore the midline electrodes were chosen as ROI. EPN has a lateral parietal distribution, so the parietal ROI was chosen. Each individual electrode within the ROI contributed a variable in ANOVA so that both the main effect of condition and the condition x electrode interaction were relevant to the testing of hypotheses. Greenhouse-Geisser correction was applied to adjust degrees of freedom for the main effect of electrode and the condition x electrode interaction. Main effects of the electrode position variable (which was not relevant to the experimental hypotheses) and non-significant results are omitted from the tables for brevity.

## Results

### Overall response to facial expression image pairs

**Fig 1:**
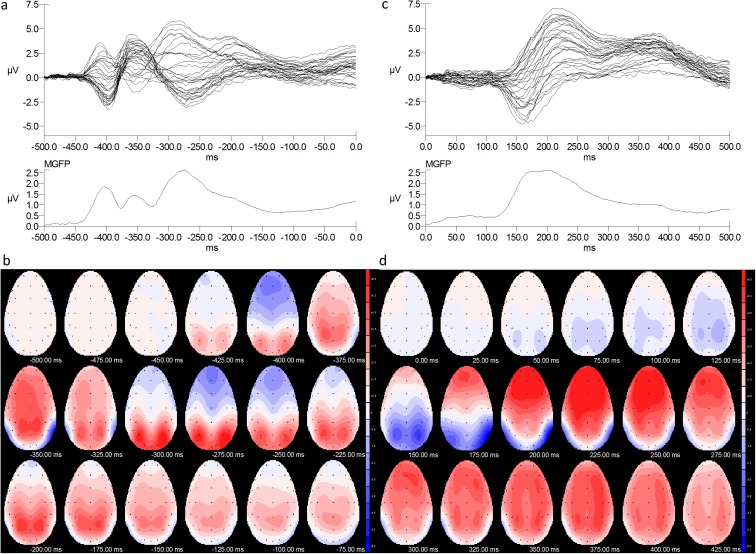
Comparison of ERP responses to onset of an angry face and transition from neutral to angry expression. Butterfly and mean global field power (MGFP) plots are shown at the top of the figure for angry face onset at -500 ms (a) and neutral-angry transitions at 0 ms (c). The corresponding ERP scalp distributions are shown in the lower half of the figure, sampled at 25 ms intervals from onset of an angry face (b) or from the transition from neutral to angry (d).

Butterfly plots and mean global field power (MGFP) plots showed a triphasic response to onset of an angry face (Fig 1A) and a biphasic response to face transition from neutral to angry (Fig 1B). Scalp distributions of ERPs to angry faces differed for face onset (Fig 1C) and transition from neutral (Fig 1D). One of the differences was the presence of a clear posterior P1 at 75–100 ms for face onset (fourth icon, Fig 1C) and a reduced P1 for face transition (fourth icon, Fig 1D). Another difference was the presence of a vertex positive potential (VPP) accompanying posterior N1 for face onset: this was delayed and not distinct from P2 for neutral-angry transition. A third difference was that the cortical minima for the N170 to face onset were at P8 and P7 and for the N1 to face transition they were at P4 and P3. At around 250 ms onwards face onset produced a positive wave on posterior and a negative wave on anterior electrodes. This late anterior negativity to face onset stimuli has been characterised as N3 and linked to late stages of expression categorisation [31].

For face transition, this pattern was inverted, with the N1 at 150–200 ms giving way to a lateralised parietal negativity labelled EPN [18] and a strong frontal-central positivity at 175–275 ms which we have labelled as P2, giving way to a late positive complex, LPC [18]. One further point to note is that the response to face onset has diminished but not ended at 0 ms (500 ms after face onset) at which time the baseline was reset to record the response to transition. The data presented in Fig 1 suggest that the ERP response to face onset and face transition is qualitatively different. The main focus of this paper will be the response to transitions, but statistical analysis of responses to face onset will also be briefly summarised.

### Experiment 1: Comparison of responses to changes in emotional expression and changes in magnification

#### 1.1 Behavioural results

During ERP acquisition, participants indicated whether trials involved a change in emotional expression or a change in magnification (control condition) by pressing a key. Sensitivity is calculated as true positives / true positives + false negatives [32] thus for the stimulus “angry-neutral” the response “emotion change” was a true positive, and the response “magnification change” to the stimulus “angry-neutral” was a false negative, and so on. Mean (*SD*) of sensitivity (expressed as percentage) was 97.2 (4.8) for neutral-angry; 97.5 (3.5) for neutral-magnified; 95.4 (7.7) for angry-neutral; and 97.6 (3.9) for magnified-neutral trials. Scores showed a ceiling effect, and Kolmogorov-Smirnov tests showed a significant departure from normality (*p* < .05) for neutral-magnified trials, so nonparametric tests were used for data analysis. Paired comparisons showed a significant difference in sensitivity between neutral-angry and angry-neutral (Wilcoxon *T =* 18, *N* = 19, *p* < .05). Other paired comparisons were not significant. It is plausible that a looming face might be perceived as threatening and a receding face as fearful, however there was no tendency to classify magnification change as emotional: false positives for magnification change were not significantly different from false positives for expression change, (Wilcoxon *T* = 87, *N* = 19, *p* = .35). Moreover in all four conditions, expression change was consistently differentiated from magnification change at better than chance (50%) level (one sample Wilcoxon versus 50% median, all *p* < .0005). Because of the small numbers of errors, for the purposes of ERP measurement only trials with correct identification of targets were analysed.

#### 1.2. Mapping of ERP differences between experimental conditions

**Fig 2:**
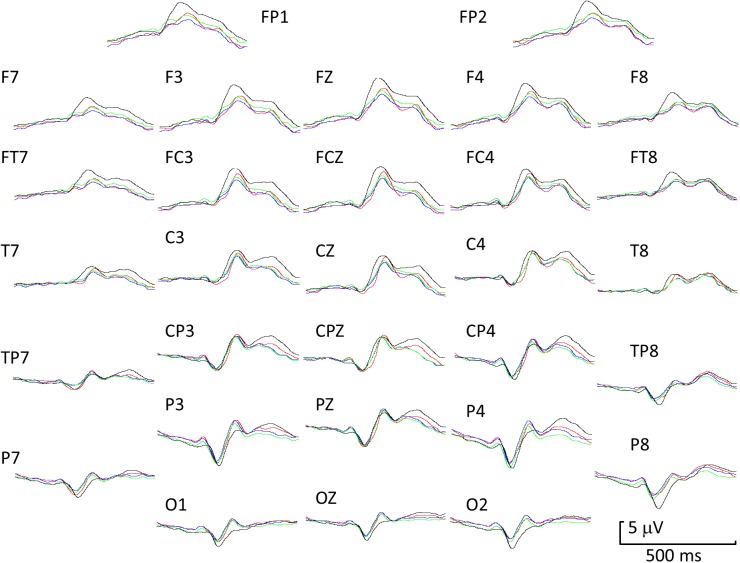
Grand mean ERP waveforms for the response to the transition in a face pair for all four experimental conditions: black = neutral-angry, red = neutral-magnified, green = angry-neutral, blue = magnified-neutral. Positive is upwards.

Fig 2 shows grand averaged ERPs to all four transitions across all electrodes: The general description of waveforms comprises a very small P1, a prominent N1 (at around the N170 latency), a clear P2 peak particularly for the neutral-angry condition over frontal electrodes, an EPN visible on central and parietal electrodes and a late LPC forming a distinct peak in some conditions.

**Fig 3:**
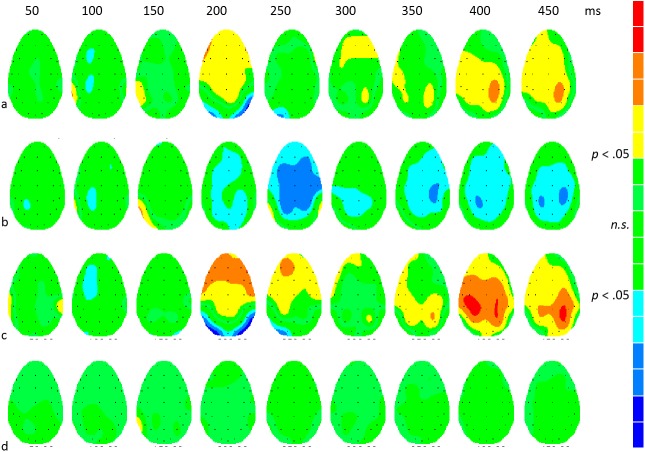
*t*-maps showing the scalp distribution of significant differences between experimental conditions. The colour scales have been adjusted for each comparison so that *t*-values generating non-significant differences (FDR-corrected *p* > .05) are shown as green. Significant *t-*values are shown as yellow, orange and red for positive differences and cyan, light and dark blue for negative differences. a: neutral-angry minus neutral-magnified (*t crit* = ± 3.4); b: angry-neutral minus magnified-neutral (*t crit* = ± 2.7); c: neutral-angry minus angry-neutral (*t crit* = ± 2.9); d: neutral-magnified minus magnified-neutral (all *n*.*s*.).

Fig 3 shows differences between experimental conditions based on within-participant *t*-tests of the differences between ERPs to face transitions. The significance levels have been adjusted with a correction for false discovery rates. The results showed significant differences for the comparison of neutral-angry with neutral-magnified transitions (Fig 3A). There were smaller but significant differences in the response to the angry-neutral transition when compared with the magnified-neutral transition (Fig 3B). These are of the opposite sign to those in Fig 3A. The response to neutral-angry was significantly more positive in voltage than to angry-neutral (Fig 3C). The main onset of significant differences in Fig 3A, 3B and 3C appeared at 200 ms, although there were weak early effects. Significant differences were also found at 300 ms and later. However the comparison of neutral-magnified with magnified-neutral yielded no significant effects (Fig 3D).

#### 1.3 ROI analysis of expression change versus magnification change

A separate ANOVA was carried out for each hypothesis and each temporal window, the independent variables being condition (two levels) and electrode position (five or six levels) within the ROI (region of interest). The dependent variables of peak amplitude and peak latency were analysed separately. The main effect of electrode position within a ROI was tested in ANOVA, but is not reported for the sake of brevity, since it has no bearing on the experimental hypotheses. On the other hand, both the main effects of experimental condition, and the condition x electrode interaction are reported as they are pertinent to the experimental hypotheses. Only statistically significant effects are included in the tables.

**Hypothesis 1.1** was tested by a comparison between neutral-angry and neutral-magnified transitions. Table 1 summarises significant effects for tests of H1.1 for the preselected regions of interest.

**Table 1:** 

Hypothesis 1.1 Neutral-angry versus neutral-magnified						Mean	
component	factor	*F*	*df*	*p*	η^2^_p_	N_A	N_M
N1 parietal amplitude	Condition x electrode	5.98	2.50, 44.9	< .005	.25		
N1 parietal latency	Condition	14.4	1,18	< .005	.44	162 ms	153 ms
P2 midline amplitude	Condition x electrode	6.82	2.13, 38.4	< .005	.28		

Both univariate (Fig 3A) and ROI results support H1.1, in that there are differences in ERPs to transitions from a neutral to an emotional expression and from neutral to a matched magnification change.

**Hypothesis 1.2** is based on a comparison between angry-neutral and magnified-neutral transitions in ROIs. A summary of the ANOVA results is given in Table 2.

**Table 2:** 

Hypothesis 1.2 Angry-neutral vs magnified-neutral						Mean	
component	factor	*F*	*df*	*p*	η^2^_p_	A_N	M_N
P1 occipital latency	Condition	6.0	1,18	< .05	.25	104 ms	97.6 ms
N1 parietal latency	Condition	30.6	1,18	< .0005	.63	164 ms	155 ms
P2 midline amplitude	Condition	8.18	1,18	< .05	.31	2.65 μV	4.75 μV
P2 midline latency	Condition	4.68	1,18	< .05	.21	233 ms	226 ms
EPN parietal amplitude	Condition	4.89	1,18	< .05	.21	-1.02 μV	-.22 μV
LPC midline amplitude	Condition	6.87	1,18	< .05	.28	1.85 μV	3.54 μV
LPC midline amplitude	Condition x electrode	3.36	2.33, 41.8	< .05	.16		

Both univariate (Fig 3B) and ROI results support H1.2, in that there are differences in the ERP to a transition to neutral face according to whether the preceding face is an angry or a magnified neutral face.

#### 1.4. ROI analysis of direction of change

**Hypothesis 1.3** proposed an asymmetric response to transitions between neutral and angry facial expressions. The results of ANOVA are summarised in Table 3. Both univariate (Fig 3C) and ROI results support H1.3, confirming a directional asymmetry in the response to transitions between angry and neutral faces, with larger ERPs to neutral-angry than to angry-neutral.

**Table 3:** 

Hypothesis 1.3 Neutral-angry versus angry-neutral						Mean	
component	factor	*F*	*df*	*p*	η^2^_p_	N_A	A_N
N1 parietal amplitude	Condition x electrode	5.91	2.32, 41.7	< .005	.25		
N1 parietal latency	Condition x electrode	3.14	3.11, 56.0	< .05	.15		
P2 midline amplitude	Condition	18.6	1,18	< .0005	.51	5.6 μV	2.7 μV
P2 midline amplitude	Condition x electrode	18.7	2.87, 51.7	< .0005	.51		
P2 midline latency	Condition	8.62	1,18	< .05	.32	226 ms	233 ms
EPN parietal amplitude	Condition	5.20	1,18	< .05	.22	.18 μV	-1.0 μV
EPN parietal amplitude	Condition x electrode	7.70	2.67, 48.1	< .0005	.30		
LPC midline amplitude	Condition	15.8	1,18	< .005	.47	5.0 μV	1.9 μV
LPC midline amplitude	Condition x electrode	4.5	2.69, 48.3	< .05	.20		
LPC midline latency	Condition	9.22	1,19	< .05	.33	364 ms	337 ms

**Hypothesis 1.4** proposed an asymmetry in the response to a looming versus a receding neutral face image. Although there were apparent differences in the ERP waveforms and the statistical parametric scalp maps (*t-*values), none of these differences survived the FDR correction for multiple comparisons (Fig 3D). However the comparison of differences between conditions using ANOVA in pre-selected regions of interest yielded some significant effects (Table 4).

**Table 4:** 

Hypothesis 1.4 Neutral-magnified versus magnified-neutral						Mean	
component	factor	*F*	*df*	*p*	η^2^_p_	N_M	N_M
N1 parietal amplitude	Condition	4.65	1,18	< .05	.21	-4.69 μV	-4.14 μV
N1 parietal amplitude	Condition x electrode	4.67	2.99, 53.8	< .005	.21		
EPN parietal latency	Condition x electrode	2.90	2.94, 53.0	< .05	.14		

Overall, evidence for directional asymmetries in transitions between neutral and magnified faces (H1.4) is mixed. In the FDR-corrected SPMs (Fig 3D) there were no significant asymmetries. However ANOVA in regions of interest identified significant differences in the amplitudes of N1 peaks which in view of the inconsistency between the different statistical tests will require further corroboration.

#### 1.5. Responses to face onset: Supplementary findings

Differences between the onset of an angry face and a neutral face were found in the N1 (N170) range across the parietal region of interest. The mean amplitude for the onset of neutral stimuli was more negative (*M* = -0.91) than for angry stimuli (*M* = -0.41), *F*(1,18) = 5.24; *p* = .05; η^2^_p_ = .23. For the P2 midline ROI there was a significant main effect of condition, with a larger amplitude for angry onsets (*M* = 4.2) than for neutral onsets (*M* = 3.4), *F*(1,18) = 5.11, p < .05, η^2^_p_ = .22; and a significant interaction between condition (angry versus neutral onset) and electrode position, *F*(2.66, 47.9) = 3.26; *p* < .05; η^2^_p_ = .15. All other comparisons gave non-significant differences.

### Experiment 2: Comparison of responses to changes in happy and frightened facial expressions

The second experiment set out to compare transitions involving facial expressions of two different emotions, happiness and fear, rather than comparing an emotional change with a non-emotional change.

#### 2.1. Behavioural results

During EEG acquisition participants indicated whether a trial included a happy or a frightened face by pressing a key. Sensitivity was calculated as true positives / true positives + false negatives [32], thus for the stimulus “happy-neutral” the response “happy” was a true positive, and the response “frightened” to the stimulus “happy-neutral” was a false negative, and so on. Mean (*SD*) of sensitivity (expressed as percentage) was 96.7 (4.9) for neutral-happy; 94.7 (5.6) for neutral-frightened; 89.1 (11.8) for happy-neutral and 90.9 (10.1) for frightened-neutral. Exploratory tests showed a significant departure from normality of distribution for neutral-happy (Kolmogorov-Smirnov *p* < 0.05) so nonparametric tests were employed. Wilcoxon matched-pairs, signed ranks showed a significant effect or order: for neutral-happy versus happy-neutral, Wilcoxon *T* = 10, *N* = 16, *p* < .005; and for neutral-frightened versus frightened-neutral, Wilcoxon *T* = 14, *N* = 16, *p* < .01. Sensitivity was greater when the emotional expression followed the neutral expression. Also sensitivity was higher for happy-neutral than for frightened-neutral, Wilcoxon *T* = 16, *N* = 16, *p* < .05. Comparing happy-neutral with frightened-neutral there was no significant difference. Because of relatively small numbers of errors, only trials with correct responses were used for ERP analysis.

#### 2.2. Mapping of ERP differences between experimental conditions

**Fig 4:**
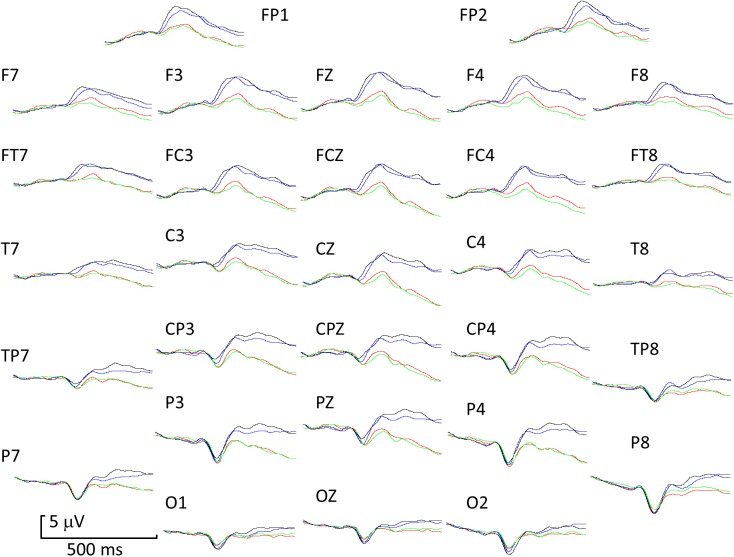
Grand-average ERP waveforms transitions between neutral and happy or neutral and frightened facial expressions. Blue trace = neutral-frightened, black trace = neutral-happy, red trace = frightened-neutral, green trace = happy-neutral. Positive upwards.

Fig 4 shows grand averaged ERPs to all four transitions across all electrodes: The general description of waveforms comprises a very small P1, a prominent N1 (minimum at around the N170 latency), a clear P2 peak particularly for the neutral-angry condition over frontal electrodes, an EPN visible on central and parietal electrodes and a late LPC forming a distinct peak in some conditions. The hypotheses tested were firstly that there were differences in ERP related to the specific emotion expressed in the transitional change, and secondly sensitivity to the transitions depended on the direction of change.

**Fig 5:**
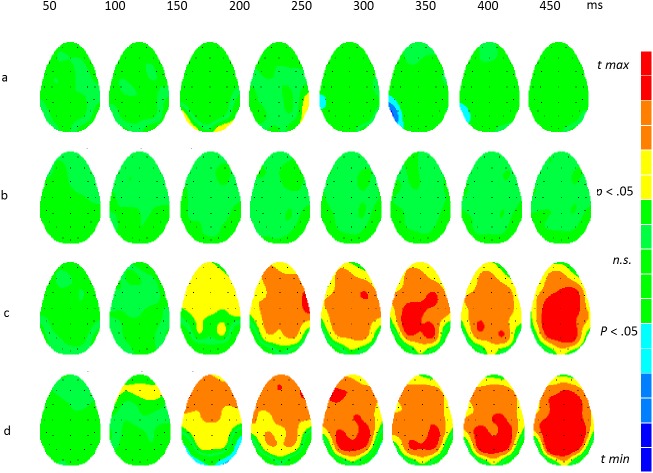
*t-*maps showing differences between experimental conditions. The colour scales have been adjusted for each comparison so that *t*-values generating non-significant differences (FDR-corrected *p* > .05) are shown as green. Significant *t-*values are shown as yellow, orange and red for positive differences and cyan, light and dark blue for negative differences. a. neutral-frightened minus neutral-happy (*t crit* = ± 3.9); b. frightened-neutral minus happy-neutral, (all *n*.*s*); c. neutral-frightened minus frightened-neutral (*t crit* = ± 2.4); d. neutral-happy minus happy-neutral (*t crit* = ± 2.3).

Fig 5 shows the locus on the scalp electrode array of statistically significant differences between experimental conditions. The colour scales in Fig 5 were adjusted separately for each comparison such that *t-*values generating non-significant differences (FDR-corrected *p* > .05) are always shown as green. Positive differences between conditions give rise to positive *t-*values (FDR corrected *p* < .05 yellow and red) and negative differences yield negative *t-*values (FDR corrected *p* < .05 cyan and blue). The SPMs were sampled every 50 ms from 50 (first column) to 450 ms (last column) after transition. When neutral-frightened and neutral-happy transitions were compared, localised differences around lateral parietal electrodes survived the FDR correction (Fig 5A). Fig 5B showed no significant differences for frightened-neutral versus happy-neutral; the third row (Fig 5C) reveals widespread differences across the scalp from 200 ms onwards, for neutral-frightened versus frightened-neutral plus early differences in right lateral occipital and parietal cortex. Finally, the comparison of neutral-happy versus happy-neutral (Fig 5D) showed widespread differences from 150 ms onwards.

#### 2.3. A comparison of ERPs to transitions involving happy versus frightened facial expressions; ROI results

**Hypothesis 2.1** proposed that there would be significant differences in transitions that were specific to the emotion. Table 5 shows significant findings from tests of the hypothesis that the ERP to a change from neutral differs for frightened and happy expressions.

**Table 5:** 

Hypothesis 2.1 Neutral-frightened versus neutral-happy						Mean	
component	factor	*F*	*df*	*p*	η^2^_p_	N_F	N_H
N1 parietal amplitude	Condition	4.88	1,15	< .05	.26	-3.68	-3.14
N1 parietal amplitude	Condition x electrode	3.94	2.44, 36.5	< .05	.21		
P2 midline amplitude	Condition	7.44	1,15	< .05	.33	4.16 μV	4.98 μV
EPN parietal amplitude	Condition	22.3	1,15	< .0005	.60	-.97 μV	-.08 μV
LPC midline amplitude	Condition x electrode	3.28	2.11, 31.7	< .05	.18		

In support of H2.1 there were significant differences in ERP to neutral-frightened and neutral-happy transitions, and these differences were evident in the amplitudes and latencies of N1 peaks and the amplitudes of P2 and LPC peaks (Table 5). The comparison of neutral-frightened versus neutral-happy transitions in *t*-maps (Fig 5A) shows that spatially the differences were concentrated in lateral occipital and parietal locations, greater positivity for the frightened transition appeared early (50–200 ms) over right hemisphere and this was followed by greater positivity for the happy transition at 300–350 ms. However the differences in the P1 latency range in Fig 5A were not confirmed by ANOVA.

**Hypothesis 2.2.** A comparison between frightened-neutral and happy-neutral transitions tests the hypothesis that the ERP to a transition to a neutral expression depends on the initial emotional expression. If the ERPs measured are reflecting a true dynamic process, rather than the separate response to two static face images, then the response should depend on the initial state as much as on the final state. Statistical parametric maps (paired *t-*scores) were calculated to test the distribution of the differences across the scalp. None of these differences survived the correction for false discovery rate (Fig 5B). Using a more sensitive method based on predefined regions of interest, ANOVA tests were conducted to identify significant changes in peak amplitude and latency of waveforms within pre-selected electrode clusters and sampling intervals. N1 amplitudes following a frightened face were significantly larger (more negative) than following a happy face. However significant effects on a parietal ROI were found in a post-hoc analysis as reported in Table 6.

**Table 6:** 

Hypothesis 2.2 Frightened-neutral versus happy-neutral						Mean	
component	factor	*F*	*df*	*p*	η^2^_p_	F_N	H_N
N1 parietal amplitude	Condition	5.37	1,15	< .05	.26	-2.67 μV	-2.01 μV
P2 parietal amplitude*	Condition x electrode	4.22	2.73, 43.7	< .05	.21		

* post-hoc ROI

Thus there was no strict confirmation for H2:2. The ROI test indicates there were subtle localised effects that require further investigation.

#### 2.4. Direction of change in emotional expressions

**Hypothesis 2.3.** The comparison of ERPs to frightened-neutral and neutral-frightened transitions tests the hypothesis that responses to an expression change are asymmetric for different directions of change. Statistical parametric scalp maps based on paired-sample *t-*tests (Fig 5C) showed that there are significant differences in lateral parietal and occipital regions across N1 and EPN and in frontal as well as parietal-occipital across P2, and in central, frontal and parietal regions across LPC. The results of ANOVA on peak amplitude and latency of the ERP within pre-selected electrode clusters and time windows are summarised in Table 7. These results showed that for transitions between frightened and neutral expressions, the ERP depended on the direction of the change.

**Table 7:** 

Hypothesis 2.3 Neutral-frightened versus frightened-neutral						Mean	
component	factor	*F*	*df*	*p*	η^2^_p_	N_F	F_N
P1 occipital amplitude	Condition	14.46	1,15	< .005	.48	-.37 μV	.43 μV
N1 parietal amplitude	Condition	11.25	1,15	< .005	.33	-3.68 μV	-2.67 μV
N1 parietal latency	Condition	8.79	1,15	< .05	.37	165 ms	171 ms
P2 midline amplitude	Condition x electrode	9.12	1.92, 28.7	< .005	.38		
EPN parietal amplitude	Condition	34.1	1,15	< .0005	.70	-.97 μV	-3.0 μV
EPN parietal amplitude	Condition x electrode	13.2	2.03, 30.5	< .005	.47		
P3 midline amplitude	Condition	8.64	1,15	< .05	.37	3.55 μV	1.79 μV
LPC midline amplitude	Condition x electrode	4.78	2.24, 33.7	< .05	.24		
LPC midline latency	Condition	7.29	1,15	< .05	.38	359 ms	326 ms

**Hypothesis 2.4.** The comparison of happy-neutral with neutral-happy transitions tests the hypothesis that ERPs depend on the timewise symmetry of the change (H2.4). Statistical parametric scalp maps based on paired-sample *t-*tests (Fig 5D) showed results similar to those obtained with transitions between neutral and frightened expressions. Significant differences appeared at 150 ms or earlier over frontal electrodes. There were significant differences in frontal, lateral parietal and occipital regions from 200 ms, and in central, frontal and parietal regions from 300 ms. The comparison of differences between conditions using ANOVA in ROIs yielded some significant effects (Table 8).

**Table 8:** 

Hypothesis 2.4 Neutral-happy versus happy-neutral						Mean	
component	factor	*F*	*df*	*p*	η^2^_p_	N_H	H_N
P1 occipital amplitude	condition	21.7	1,16	< .005	.58	-.34 μV	.40 μV
N1 parietal amplitude	Condition	11.3	1,15	< .005	.43	-3.8 μV	-2.5 μV
N1 parietal latency	Condition	4.74	1,15	< .05	.29	163 ms	171 ms
N1 parietal latency	Condition x electrode	6.03	3.03. 35.4	< .005	.29		
P2 midline amplitude	Condition	9.79	1,15	< .05	.38	4.98 μV	3.46 μV
P2 midline amplitude	Condition x electrode	11.5	2.17, 32.5	< .0005	.43		
P2 midline latency	Condition	6.70	1,15	< .05	.31	236 ms	228 ms
EPN parietal amplitude	Condition	24.5	1,15	< .0005	.62	-.084 μV	-2.74 μV
EPN parietal amplitude	Condition x electrode	15.4	2.86, 42.8	< .0005	.51		
LPC midline amplitude	Condition	20.1	1,15	< .0005	.57	4.54 μV	1.79 μV
LPC midline amplitude	Condition x electrode	15.3	2.3, 34.5	< .0005	.51		
LPC midline latency	Condition	6.07	1,15	< .05	.29	356 ms	330 ms

Overall, the results show that for transitions between neutral and happy facial expressions, the ERP depends on the direction of the change (H2.4).

#### 2.5. Responses to face onset: Supplementary findings

ANOVAs were carried out on peak amplitudes to the onset of the face in the first interval, using the same temporal and spatial ROIs as for transitions. The only significant differences in ERPs between experimental conditions were found for P1. There was a significant difference in P1 amplitude over the parietal-occipital ROI to onset of frightened (*M* = 3.04 μV) and happy (*M* = 2.75 μV) faces, *F*(1,15) = 6.13, *p* < .05, η^2^_p_ = .29. For onset of happy versus neutral faces, P1 showed a significant interaction with electrode position within the ROI, *F*(2.68, 40.3) = 4.20, *p* < .05, η^2^_p_ = .22.

## Discussion

The purpose of the study was to investigate differences in event-related potentials (ERPs) between emotional and neutral facial expressions using two-phase transitions and to test whether the resulting ERPs differed for opposite directions of change. Using dynamic face expressions allowed us to investigate the transitions from neutral to emotional and from emotional to neutral expressions with an inbuilt control for other emotion-irrelevant face-processing variables. The present findings suggested that transitions from neutral to angry expressions produced more prominent ERP responses than did an increase in a control-condition of magnification. ERPs to neutral following angry, relative to neutral following magnified, were generally more negative. Furthermore, the second experiment demonstrated differences in ERPs to changes in happy and frightened expressions even though behavioural data gathered during EEG showed no significant difference in sensitivity to happy and frightened transitions.

### Comparisons of ERPs to dynamic changes in different emotional expressions

Experiment 1 showed an enhanced N1 wave for transitions from a neutral to an emotional (angry) face relative to non-emotional control changes. This negative wave had a minimum in the N170 range (150–200 ms) but instead of a vertex VPP simultaneous with N1 there was a frontal P2 at 175–300 ms (compare Fig 1D and 1B). The location of the minima on the scalp also differed for face onset and face transition (Fig 1B versus 1D), and between the early (N1) and later (EPN) part of the negative wave for face transition (Fig 1D). Also, the peak latency of the N1 wave was delayed by 10 ms at P8 and O2 in the emotional relative to the non-emotional transition (Fig 2). ERP differences were not limited to N1 but also occurred for P2. Persistent differences in the LPC range are visible in the FDR-corrected *t*-maps and this is consistent with enhancement of the late positive potentials (LPC) for dynamic emotional faces [33]. Frontal P2 and was greatest for neutral-angry transitions (Fig 2). In summary, the ERP N1-P2 amplitude to change from a neutral to an emotional face was greater than ERP amplitude to change from a neutral face to a magnified but neutral face, suggesting enhanced processing when an emotional face followed a neutral face.

Peak amplitude and latency differences were also observed in the comparison of angry-neutral and magnified-neutral transitions. Peak latency differences were similar to those observed for neutral-angry versus neutral-magnified transitions. Thus, the N1 peak for the angry-neutral transition was delayed by 10 ms relative to the magnified-neutral N1. Amplitude changes however were opposite in sign. Whereas P2 amplitudes to angry following neutral were more positive than for magnified following neutral, the amplitudes to a neutral face following an angry face were less positive than those to a neutral face following a neutral but magnified face, and differences were negative (Fig 3). This is clear evidence that both the pre- and post- transition stimuli determine the ERP to the transition.

Experiment 2 compared ERP responses to different dynamic emotional expressions. Transitions from neutral to happy and from neutral to frightened expressions produced distinct ERP profiles. Significant differences in both ROI analysis and FDR-corrected *t*-maps occurred for N1 and later for EPN. Generally, neutral-happy was processed faster than neutral-frightened and this was already evident during N1. Neutral-happy also elicited larger ERP amplitudes than neutral-frightened in parietal regions for EPN. These results are consistent with previous findings of happiness superiority in faces [34, 35]. These ERP differences could reflect differences in intensity or arousal to the stimuli rather than categorical emotion specificity [18].

If specificity to different dynamic emotional facial expressions occurs regardless of the direction of the change, then differences might be expected for happy-neutral versus frightened-neutral. No significant differences were found on FDR-corrected *t*-tests, although a significant difference was found on N1 in the ROI analysis. Conversely, in Experiment 1, comparing angry-neutral with magnified-neutral, ANOVA indicated significant effects on P1 latency, N1 amplitude, P2 latency and amplitude and on LPC amplitude; and correspondingly, FDR-corrected *t*-maps indicated that the ERP to neutral following angry was shifted negatively over a long interval relative to neutral following magnified. Despite the second phase image being identical for angry-neutral and magnified-neutral, effects of the transition from the first phase persisted in the late components (LPC) of the response to the transition. However this long-lasting effect was not evident in Experiment 2. If this shift was similar to an expression-specific adaptation aftereffect, [36–40], then it might be expected to occur for happy-neutral versus frightened-neutral. This ERP difference was simply too small to survive FDR correction in the present experiments. One possible explanation for the difference in the outcome of the two experiments is that facial expression intensity in the first interval affects the ERP on transition to neutral. On intensity ratings of the stimuli, angry and neutral differed more than happy and frightened, whereas on valence ratings, they differed less (S1 File). Therefore the ERP for transitions to neutral may be related to intensity change rather than valence or category change [18, 41].

### Comparisons of ERP to different directions of change in emotional facial expression

The direction of change (neutral to emotional or emotional to neutral) had a profound effect on the ERP to dynamic change in facial expression. Thus, in Experiment 1, (neutral-angry versus angry-neutral) for N1, P2, EPN and LPC, asymmetries were found whereas P1 was not influenced by the direction of change. N1 amplitudes were more negative and latencies shorter on P8 for neutral-angry than for angry-neutral. N1 amplitudes were more negative on electrodes P7 and P3 for neutral-magnified than for magnified-neutral. P2 amplitudes were substantially more positive and peak latencies shorter for neutral-angry than for angry-neutral, especially on frontal electrodes. LPC amplitudes were greater and the peak amplitude was later for neutral-angry than for angry-neutral. Similarly, in Experiment 2, enhanced ERPs were found for the neutral-frightened transition relative to frightened-neutral, and for neutral-happy relative to happy-neutral. An asymmetry was also noted in behavioural responses for within-session expression identification errors to dynamic stimuli. Directional results for ERP responses to transitions to and from neutral were reported by Miki et al. [20] and the present study partially replicates and extends their findings. They found N1 asymmetries for angry transitions in children but not adults whereas the present study (with a larger sample of slightly younger adults) found clearly asymmetric responses in adults for angry as well as for happy and fearful transitions.

We need to consider the possibility of confounds between responses to the direction of change and differences in the initial and/or final emotional expression–whether a target expression appears first or last. No study to date has completely solved this issue. The approach taken by Miki, et al. [20] was to use an incidental task to reduce attention to the emotional expression. However the same issue of relative timing arises for covert processing. In the current experiment, it was a priority to ensure that the emotional content was fully processed, and the need to separate the ERP to transition from preparation of the motor response was addressed by delaying the response. Perhaps the strongest indication that we are dealing with the ERP response to the transition itself, rather than the response to the second stimulus phase is seen in Experiment 1, where significant differences were found during the identical neutral phase of the second interval, due to an emotional versus a neutral image in the first phase.

The ERP to the face onset stimulus showed some significant differences between emotional and neutral faces. There was not always a clear P1 peak for transitions, but the maximum amplitude in the P1 range was more positive for transitions from emotional to neutral whereas all later waveforms showed enhancement for transitions from neutral to emotional.

Experiments in which participants rated the intensity and valence of the dynamic stimuli used in the ERP experiments (S1 File) gave results entirely consistent with the asymmetry observed in ERPs. Thus neutral-happy was rated as significantly more intense and more positive in valence than happy-neutral, neutral-frightened was rated more intense and more negative in valence than frightened-neutral and neutral-angry was also rated as more intense and more negative in valence than angry-neutral. No corresponding differences were seen for changes in magnification.

The simplest explanation for the enhancement of N1 and later components is that emotional expressions recruit more processing resources than neutral expressions. Thus, for the neutral-emotional transition these resources are increased (increased ERP amplitude), and for the emotional-neutral transition they are reduced (decreased ERP amplitude). Current findings are also consistent with adaptation studies [38–40] suggesting that facial expressions are encoded in a multidimensional “face expression space” [42] in which the neutral point represents an average expression, and where deviations from this neutral point are neurally encoded.

The sensitivity to directional expression change observed in experiment 1 and 2 is also in line with neuroimaging data showing parts of the amygdala [43] and FFA [44] to be sensitive to the direction of changes. The fMRI results also showed directional effects in STS [44] although the pattern of results was more complex.

Finally, there was an interesting incidental finding in Experiment 1. Larger ERP amplitudes were found for neutral-magnified relative to magnified-neutral in the N1 and P2 peaks in pre-selected ROIs. The neutral-magnified transition is readily seen as a movement of the face towards the observer and magnified-neutral as a movement away [45] and behavioural studies show faster recognition for approaching faces [46]. The present findings are consistent with a MEG study by Yang, Hsei and Chang [47], who found increased M160 amplitudes for expanding checkerboard stimuli in right occipital-temporal locations. The present study is first to show this ERP effect with ecologically more valid stimuli such as faces.

## Conclusions

The main aims of the present study were, firstly, to analyse the differences in ERP response to emotional expression change versus non-expressive change as well as the differences in expression change ERPs for different types of emotion expressions. Secondly, the study addressed the directionality of the ERP response in transitions from emotional to neutral versus neutral to emotional facial expressions. The present study utilised dynamic, two-phase transitions instead of commonly used static images of emotional faces in order to control for the brains’ initial response to the sudden appearance of a face regardless of its emotional expression. The benefit of using these dynamic two-phase stimuli is the elimination of emotion-irrelevant variables such as identity or low-level visual features which allows us to focus on pure emotion-induced changes. Overall, the present data suggested that the direction of change from emotional to neutral or neutral to emotional expressions can indeed influence the ERP signal and this is true for a range of emotions and is qualitatively and quantitatively different to the response to a non-emotional change in magnification of faces. Further, the change from neutral to emotional faces elicited specific ERP responses, depending on the specific emotion displayed. The predominant effects were on ERP amplitudes, suggesting that the differences are due to differing intensities of expression stimuli. However, some subtle differences in scalp distribution and peak latencies were observed which may reflect some category-specific or valence-specific effects. An important observation was that the response to a transition to neutral depends on whether the preceding face is emotional (angry) or not emotional (neutral and magnified) although there appears to be no difference in the response to neutral following two emotional expressions that are very different in emotional valence (happy, frightened). Asymmetries in the response to neutral-emotional versus emotional-neutral transitions were consistent with previous research on adaptation to facial expressions, but further experiments would be needed to demonstrate whether adaptation occurs within the brief exposures used in the present study. Generally, the present findings support face processing models [6, 11] which postulate different neural pathways for processing of stable features such as identity versus dynamic analysis of expressions and facial movements.

## Supporting information

S1 File Calibration of stimuli used in the study. (DOCX)
