# Supplementary material for: Event-related potentials to changes in facial expression in two-phase transitions
Source: PLoS One. 2017 Apr 13;12(4):e0175631. doi: 10.1371/journal.pone.0175631 (PMC5391024; doi:10.1371/journal.pone.0175631)
Supplement: S1 File — (DOCX) [file pone.0175631.s001.docx]

Supplementary file S1.

# Validation of stimuli used in the EEG study.

*External validity of source images.*

We first consider the external validity of the JAFFE [21] stationary source images. Face stimuli from two separate databases were categorized (single-interval, four-alternative forced-choice) and rated singly for valence and arousal (seven point scale) by a multi-ethnic sample (19 participants; M age 18.9; 19 female) separate from those in the main EEG study. Scores for JAFFE stimuli [21] were compared with those for female faces in the validated Ekman and Friesen (EF) dataset [22]. Stimuli were static pictures presented for 2 s.

*Accuracy.* Target emotions in the JAFFE set were not equally recognizable: Mean percent correct identifications were *M* angry = 77%, frightened 84%, happy 95%, neutral 89%. The corresponding figures for the EF dataset were: *M* angry = 93%, frightened 94%, happy 97% and neutral 87%. Paired *t*-tests indicated that the differences for accuracy between EF and JAFFE were statistically significant for anger, *t*(18) = 5.4; *p* < .0005, and fear, *t(*18) = 5.8; *p* < .0005. Differences for happy and neutral expressions were not significant.

*Intensity Ratings.* The differences in sensitivity to JAFFE versus EF faces appears to be due to the lower intensity of JAFFE expressions: participants’ mean intensity ratings of 10 JAFFE faces on a 7-point scale (1 = min, 7 = max) were significantly lower than their ratings of female faces for the same emotional expressions drawn from the EF dataset, *F*(1,18) = 23.3, *p <*.0005; η^2^_p_ = .57. Also, intensity ratings of happy, angry and frightened faces differed from one another, *F*(2,36) = 20.9, *p* < .0005; η^2^_p_ = .54, but the dataset x emotion interaction was not significant. Repeated contrasts showed that happy faces were rated as significantly more intense than frightened (*p* <.05) and frightened faces marginally more intense than angry (*p* =.057). The M (SD) of JAFFE intensity ratings were: happy 4.98 (.65), frightened 4.24 (.91), angry 3.57 (1.03) and neutral 1.89 (1.07). The *M* (*SD*) of JAFFE valences (7 = very positive, 1 = very negative) were: happy 5.39 (.49), frightened 2.74 (.41), angry 2.88 (.32), and neutral 3.97 (.27).

*Valence.* Differences in rated valence between JAFFE and EF pictures were not significant, *p* > .05.

We conclude that emotional expressions in the JAFFE set were judged similar in valence but less intense than the EF set. The target emotions were sufficiently identifiable for the purposes of research.

This calibration was based on static presentations of pictures, but the focus of the accompanying ERP study was on dynamic presentations of transitions between picture pairs. The question therefore arises as to whether ratings for emotional expressions are different for static versus dynamic presentation of the stimuli. Static and dynamic presentations of the same overall duration are reportedly equivalent in perceived valence, but participants have reported higher arousal ratings for dynamic relative to static expressions of the same maximum intensity [23]. This issue is addressed below.

Comparison of dynamic and static images.

*Intensity.* To validate the stimuli used in the EEG/ERP study, an independent sample of participants conducted ratings and categorization of 1s dynamic face presentations (two-state transitions with 500 ms exposure for each phase). The faces were drawn from the JAFFE set and the compound stimuli used were the same as in the main ERP experiments. Control conditions presented the same face image twice to create a 1s stationary exposure. In no case was there an inter-stimulus interval. 25 participants (age 18-21, 22 female) from a multi-ethnic student sample gave emotion intensity ratings on a four-point scale (1 = no emotion, 2 = weak emotion, 3 = moderate emotion, 4 = strong emotion). Stimuli were drawn from the angry (A) happy (H) frightened (F) and neutral (N) JAFFE sets and were combined pairwise with neutral second (A_N; H_N; F_N), neutral first (N_A; N_H; N_F) or no change (A_A; H_H; F_F). Additionally, a dynamic control stimulus was employed, which was a transition between neutral (N) and a 10% magnified neutral image (M), creating apparent motion towards (N_M) or away from (M_N) the observer. This also required a no change magnified control condition, (M_M). Descriptive statistics for all stimulus conditions are shown in Table S1.

Table S1 Mean (SE) of intensity ratings

|  | angry | magnification | happy | frightened |
| --- | --- | --- | --- | --- |
| neutral second | 2.40 (.08) | 1.22 (.05) | 2.74 (.08) | 2.62 (.08) |
| neutral first | 2.63 (.09) | 1.23 (.05) | 3.02 (.06) | 2.74 (.07) |
| no change | 2.61 (.08) | 1.31 (.06) | 3.05 (.07) | 2.88 (.06) |

From these means (Table S1), it can be seen that intensity ratings for magnification stimuli were lower than for emotion stimuli and neutral first and no change sequences give higher intensity ratings than neutral second in all cases. The statistical significance of differences in mean ratings will be tested below.

Guided by the design of the main EEG experiment, the first ANOVA consisted of two factors: *sequence* with three levels: (neutral second, neutral first and no change) and *stimulus type* with two levels (angry, magnification) (see first two columns in Table S1). There was a significant main effect of sequence, *F*(2,48) = 8.40, *p* < .005; η^2^_p_ = .26, and repeated contrasts showed that the significant difference arose because mean intensity was lower when the sequence ended in a neutral face (A­_N + M_N) rather than an angry or magnified face (N_A + N_M); *F*(1,24) = 8.6, *p* < .01; η^2^_p_ = .26. There was no significant difference between the neutral-first conditions (N_A + N_M) and the stationary control conditions (A_A + M_M), *p* > .05. Secondly, there was a significant effect of stimulus type, *F*(1,24) = 536.4, *p* < .0001; η^2^_p_ = .96, with higher mean intensity ratings for anger versus magnification. Finally, there was a significant interaction between sequence and type, *F*(2,48) = 3.39, *p* < .05, η^2^_p_ = .12. Repeated contrasts showed that this interaction arose because the direction of change affected transitions between neutral and angry more than transitions between neutral and magnified, *F*(1,24) = 5.3, *p* < .05, η^2^_p_ = .18. To correspond with the design of the ERP study, a second ANOVA compared transitions involving happy and frightened expressions. The independent variables were *sequence* (emotion first, neutral first, no change) and *emotion* (happy, frightened). There was a significant main effect of sequence, *F*(2,48) = 12.6, *p* < .0005, η^2^_p_ = .34. Repeated contrasts showed that the effect was due to differences between the neutral second and neutral first conditions, *F*(1,24) = 9.73, *p* < .005, η^2^_p_ = .29, rather than between neutral first and no change, *F*(1,24) = 3.06, n.s. Next, there was a significant main effect of the emotion type. Trials involving happy expressions were rated as more intense than trials involving frightened expressions, *F*(1,24) = 8.37, *p* < .005, η^2^_p_ = .26. Finally, the interaction between emotion and sequence was not significant; therefore the perceived intensity of happy and frightened expressions was not differentially altered as a function of dynamic or static presentations, *p* > .05.

The main conclusions from this experiment are as follows: first, the expression was seen as more intense in the neutral-emotional configuration than in the emotional-neutral configuration. However there was no significant difference in perceived intensity between neutral-emotional and a stationary presentation of the emotional face (matched for duration). Changes in magnification of a neutral facial expression did not significantly alter its perceived intensity.

*Comparison of dynamic and static sequences*

*Categorisation.* Next we consider the effects of dynamic versus static sequences on categorization. A second experiment was conducted using the same group of participants who undertook the intensity ratings (except one participant did not complete the categorization task). The same stimulus set in which the task was to categorize the main emotion present, with four alternatives provided: happy, angry, frightened and neutral. The 12 image pairs were the same as in the intensity rating experiment, but the dependent measure was the accuracy (percent of correct responses) where the correct emotion was the target emotional expression in all pairs, and where for magnification pairs the target emotion was neutral. This designation was justified in view of the low intensity ratings accorded to all magnification pairs, as reported above.

Table S2 Mean (SE) of categorization accuracy

|  | angry | magnification | happy | frightened |
| --- | --- | --- | --- | --- |
| neutral second | 62.9% (3.6%) | 90.0% (3.2%) | 76.7% (4.4%) | 66.3% (3.9%) |
| neutral first | 91.7% (4.3%) | 87.9% (3.2%) | 91.7% (1.9%) | 77.1% (3.0%) |
| no change | 85.4% (3.8%) | 90.8% (2.2%) | 85.4% (2.3%) | 76.7% (3.9%) |

The statistical significance of the differences between conditions was assessed by ANOVA. The first ANOVA compared stimuli involving angry targets with stimuli involving magnified targets. The dependent measure was accuracy of identification of the target emotion. There was a significant main effect of sequence, *F*(2,46) = 3.5, *p <*.05, η^2^_p_ = .16. Repeated contrasts showed that the effect was due to difference between the neutral second and neutral first conditions, *F*(1,23) = 4.28, *p* < .05, η^2^_p_ = .16, and that the difference between neutral first and no change conditions was not significant. There was also a significant main effect of expression type, *F*(1,23) = 16.5, *p <* .0005, η^2^_p_ = .42, with neutral target stimuli consistently more accurately identified than emotional target stimuli (Table S2). The interaction between target emotion and sequence was also significant, *F*(2,46) = 5.89, *p* < .05, η^2^_p_ = .20. Repeated contrasts showed that the target differences were significant only for comparisons between the two dynamic conditions, *F*(1,23) = 12.3, *p* < .005, η^2^_p_ = .20 not between the neutral first and control condition.

The second ANOVA compared conditions with happy or frightened target emotions. The sequence variable failed Mauchly’s test for homogeneity of variance, requiring a Greenhouse-Geisser epsilon correction (ε = .703). After correction, the main effect of sequence was significant, *F*(2,46) = 1.7, *p* < .005, η^2^_p_ = .34. Repeated contrasts showed that the effect was significant only on comparison of the two dynamic conditions, *F*(1,23) = 14.8, *p* < .0005, η^2^_p_ = .39. Differences between neutral-first transitions and no change conditions (stationary presentations) were not significant. Secondly, there was a significant main effect of emotion type (happy versus frightened), *F*(1,23) = 12.8, *p* < .005, η^2^_p_ = .36, with happy target images being more accurately identified than frightened (Table S2). The interaction of sequence and target emotion was not significant.

Taken together, results consistently showed that there were differences in the perceived emotion content of dynamic and static stimuli. The target emotion in a stimulus pair consisting of a neutral and an emotional expression was more accurately identified and rated higher in emotional intensity when the emotional image followed the neutral image than when it preceded the neutral image. Neutral-emotional pairs were not significantly different from stationary control stimuli of the same emotion and total duration, either in emotion identification or in intensity rating. Neutral stimulus pairs whether static or changing dynamically in magnification were consistently categorized as neutral and had low intensity ratings.

*Comparison of dynamic and static sequences*

*Valence*. 14 participants (age 18-21, all female) from a multi-ethnic student sample gave emotion valence ratings on a five-point scale (1 = unpleasant, 2 = mildly unpleasant, 3 = neutral, 4 = mildly pleasant, 5 = pleasant). They were instructed to rate the emotional expression, not their own feelings. Table S3 shows mean (SE) valence ratings for judgments of the emotional expressions.

Table S3 Mean (SE) ratings of valence

|  | angry | magnified | happy | fear |
| --- | --- | --- | --- | --- |
| neutral second | 2.350 (.113) | 2.907 (.049) | 3.814 (.176) | 2.236 (.114) |
| neutral first | 1.871 (.090) | 2.943 (.036) | 4.329 (.080) | 1.793 (.071) |
| no change | 1.886 (.131) | 2.957 (.042) | 4.500 (.084) | 1.743 (.095) |

To assess the statistical significance of the findings, two separate ANOVAs were conducted, in order to parallel the design of the associated ERP study. The first ANOVA used the data from valence ratings of stimuli involving angry expressions or neutral and magnified neutral images. The stimuli were identical with those in the intensity ratings and categorisation studies described above, only the task changed. The independent variables in the first ANOVA were *sequence* (neutral second, neutral first, no change) and facial *expression* (angry, neutral/magnified). There was a significant main effect of sequence, *F*(2,26) = 8.45, *p <*.005, η^2^_p_ = .39. Repeated contrasts showed that this was due to a difference between the neutral second and neutral first conditions, *F*(1,13) = 11.8, *p* < .0005, η^2^_p_ = .48, but the difference between neutral first and no change was not significant. There was a significant main effect of emotion on valence (angry versus magnified), *F*(1,13) = 91.1, *p <*.0005, η^2^_p_ = .88, and a significant interaction between sequence and emotion, *F*(2,26) = 7.6, *p <*.01, η^2^_p_ = .37. Repeated contrasts showed that this arose in the comparison of anger and magnification changes in the dynamic stimuli (neutral second vs neutral first), *F*(1,13) = 11.2, *p <*.01, η^2^_p_ = .46.

In the comparison of happy and fearful static and dynamic expressions, ANOVA revealed no significant main effect of sequence, *F*(2,26) = 1.25, n.s. As expected there was a significant main effect of valence as happy expressions were generally judged pleasant and frightened experiments as unpleasant, *F*(1,13) = 348.1, *p* < 0005, η^2^_p_ = .96. There was a significant interaction between emotion and sequence, *F*(2,26) = 12.8, *p* < .005, η^2^_p_ = .50. Repeated contrasts showed that the differences in perceived valence of happy and fearful stimuli occurred when comparing the neutral second and neutral first dynamic conditions, *F*(1,13) = 10.7, *p* < .01, η^2^_p_ = .45. Referring to the mean valence scores (table S3) for *F*_N versus N_F and H_N versus N_F it can be seen that when the emotional face was followed by a neutral face, the overall valence shifts in the direction of neutral. When the neutral face precedes the emotional face, there was no shift, and valence was judged as approximately equal to that of a stationary (no change) expression.

*Conclusions.*

Overall therefore, the results were quite straightforward. Firstly, the JAFFE faces [21] were similar in their properties (e.g. in rated valence) to the widely-used Ekman & Friesen emotional facial expression images [22], but tended to be lower in perceived intensity, with anger and fear somewhat less identifiable. Comparison of two-phase dynamic stimuli shows that the sequence of a dynamic facial expression affects ratings of intensity, and valence, and categorization accuracy. Where the emotional expression follows a neutral expression, rated intensity, rated valence and categorization were similar to that of a stationary expression of the same total duration. For a sequence where the neutral expression follows the emotional expression, rated intensity and rated valence was shifted in the direction of neutral, and categorization accuracy decreased. However these shifts were partial. Neutral stimuli involving magnification changes were not significantly affected by sequence. These results would be explained if there is a recency bias when processing a dynamic expression. The lack of differences between neutral-first dynamic and static presentations differs from some published work [23] where dynamic stimuli produced greater arousal. However arousal was not measured in the present experiments and the ratings were stimulus ratings rather than self-ratings. Another issue is on what basis dynamic and static stimuli should be matched. In the present study stimuli were matched in terms of the source images and the total duration, but that means that they were unmatched on the time-averaged expression: if the dynamic stimuli were compared to 50% neutral and 50% emotional morphed expressions, this might have given rise to an apparent superiority of the dynamic expression.
